# Matrix Metalloproteinase-12 Is Required for Granuloma Progression

**DOI:** 10.3389/fimmu.2020.553949

**Published:** 2020-09-18

**Authors:** Arjun Mohan, Nicole Neequaye, Anagha Malur, Eman Soliman, Matthew McPeek, Nancy Leffler, David Ogburn, Debra A. Tokarz, Warren Knudson, Sina A. Gharib, Lynn M. Schnapp, Barbara P. Barna, Mary Jane Thomassen

**Affiliations:** ^1^Division of Pulmonary, Critical Care and Sleep Medicine, Department of Medicine, Brody School of Medicine- East Carolina University, Greenville, NC, United States; ^2^Department of Pharmacology and Toxicology, Faculty of Pharmacy, Zagazig University, Zagazig, Egypt; ^3^Department of Population Health and Pathobiology, College of Veterinary Medicine, North Carolina State University, Raleigh, NC, United States; ^4^Department of Anatomy and Cell Biology, Brody School of Medicine- East Carolina University, Greenville, NC, United States; ^5^Division of Pulmonary, Critical Care and Sleep Medicine, Computational Medicine Core, Center for Lung Biology, Department of Medicine, University of Washington, Seattle, WA, United States; ^6^Allergy, Pulmonary and Critical Care Medicine, Department of Medicine, University of Wisconsin-Madison School of Medicine and Public Health, Madison, WI, United States

**Keywords:** sarcoidosis, MMP12, PPARγ, MWCNT, granuloma, inflammation

## Abstract

**Background:**

Sarcoidosis is a chronic inflammatory disease of unknown cause characterized by granuloma formation. Mechanisms for chronic persistence of granulomas are unknown. Matrix Metalloproteinase-12 (MMP12) degrades extracellular matrix elastin and enables infiltration of immune cells responsible for inflammation and granuloma formation. Previous studies report increased MMP12 in sarcoidosis patients and association between MMP12 expression and disease severity. We also observed elevated MMP12 in our multiwall carbon nanotube (MWCNT) murine model of granulomatous inflammation. Here we hypothesized that MMP12 is important to acute and late phases of granuloma pathogenesis. To test this hypothesis, we analyzed granulomatous and inflammatory responses of *Mmp12 knock-out* (KO) mice at 10 (acute) and 60 days (late) after MWCNT instillation.

**Methods:**

C57BL/6 (wildtype) and *Mmp12* KO mice underwent oropharyngeal instillation of MWCNT. Lungs were harvested at 3, 10, 20, and 60 days post instillation for evaluation of MMP12 expression and granulomatous changes. Bronchoalveolar lavage (BAL) cells were analyzed 60 days after MWCNT instillation for expression of mediators thought to play a role in sarcoid granulomatosis: peroxisome proliferator-activated receptor-gamma (PPARγ), interferon-gamma (IFN-γ), and CCL2 (MCP-1).

**Results:**

Pulmonary granuloma appearance at 10 days after MWCNT instillation showed no differences between wildtype and *Mmp12* KO mice. In contrast, by 60 days after MWCNT instillation, *Mmp12* KO mice revealed markedly attenuated granuloma formation together with elevated PPARγ and reduced IFNγ expression in BAL cells compared to wildtype. Unexpectedly, *Mmp12* KO mice further demonstrated increased alveolar macrophages with increased CCL2 at 60 days.

**Conclusions:**

The striking reduction of granuloma formation at day 60 in *Mmp12* KO mice suggests that MMP12 is required to maintain chronic granuloma pathophysiology. The increased PPARγ and decreased IFNγ findings suggest that these mediators also may be involved since previous studies have shown that PPARγ suppresses IFNγ and PPARγ deficiency amplifies granuloma formation. Interestingly, a role of MMP12 in granuloma resolution is also suggested by increases in both macrophage influx and CCL2. Overall, our results strongly implicate MMP12 as a key factor in granuloma persistence and as a possible therapeutic target in chronic pulmonary sarcoidosis.

## Introduction

Sarcoidosis is a prototypic granulomatous inflammatory disorder which predominantly affects the lungs and thoracic lymph nodes (1). Recent studies show that average sarcoidosis-associated mortality has increased by approximately 3% per year in the United States (2, 3). Unfortunately, large gaps in our understanding of sarcoidosis pathogenesis have hindered research and development of novel therapies. Animal models may be helpful for exploring select pathways and directing research toward higher yield mechanisms. Our multiwall carbon nanotube (MWCNT) based murine model of granulomatous inflammation was first described in 2011 (4). The model replicates human disease at multiple biological levels including key mediators such as IFN-γ and PPARγ. The transcription factor, PPARγ is a regulator of glucose and lipid metabolism but is also recognized as a negative regulator of macrophage activation (5). Alveolar macrophages from healthy individuals express constitutively high PPARγ levels but PPARγ is deficient in alveolar macrophages from sarcoid patients (6). Our previous studies with the MWCNT model indicated decreased PPARγ (7). Further studies with macrophage-specific *Ppar*γ knock out (KO) mice revealed enhanced granulomatous disease as evidenced by increased granuloma size and incidence (7). Interestingly, previous studies indicated that IFNγ represses PPARγ in human alveolar macrophages (8), suggesting a reciprocal relationship.

MMP12, also known as macrophage metalloelastase is a member of a family of extracellular endopeptidases (9, 10). MMPs were originally thought to be mainly responsible for turnover and degradation of extracellular matrix components. However, in recent years it has become clear that MMPs mediate many crucial functions in immunity and repair including cell migration, leukocyte activation and anti-microbial defense (9, 10). Furthermore, many of the earlier *in vitro* studies may not accurately reflect the *in vivo* situation (11). As Giannandrea and Parks note in their review, degradation studies with individual substrates show that isolated MMPs have redundancy *in vitro*, but *in vivo* functions of specific MMPs are limited and unique (11). MMP12 was first implicated as a mediator in sarcoidosis pathogenesis in 2009 (12). In those studies, lung tissues from sarcoidosis patients showed increased (>25-fold) *Mmp12* gene expression. Interestingly, MMP12 expression was highest near areas of active granulomatous inflammation, and MMP12 levels in bronchoalveolar fluid (BALF) correlated with disease severity. These findings make MMP12 biology an area of acute interest in sarcoidosis pathogenesis.

Using gene network analysis we previously demonstrated that MMP12 was one of the most highly expressed genes in MWCNT-exposed mice as well as in sarcoidosis patients, suggesting that MMP12 is a putative driver of granulomatous disease (detailed microarray information and raw data have been deposited in Gene Expression Omnibus^1^ [GSE 100500 and GSE75023 (13, 14)]. We therefore hypothesized that MMP12 is critical to granuloma formation. Because conclusions made from *in vitro* studies have proven to be poor predicators of *in vivo* pathogenesis, an animal model which replicates many of the features of sarcoidosis is essential. To test this hypothesis, we compared *in vivo* granuloma genesis in *Mmp12* gene KO versus wild type mice after exposure to MWCNT.

## Materials and Methods

### Multiwall Carbon Nanotube Model

All studies were conducted in conformity with Public Health Service (PHS) Policy on humane care and use of laboratory animals and were approved by the institutional animal care and use committee. C57BL/6J wild-type, *Mmp12* KO mice (Jackson Laboratories, Bar Harbor, ME, United States) and macrophage-specific *Pparγ* KO (15) received a single oropharyngeal instillation of MWCNT (100 μg) in PBS/35%surfactant (Ony Inc, Amherst, NY, United States) (4). Briefly, mice were sedated with isofluorane and by gently pulling forward the mouse tongue the epiglottis was exposed and a 50 μl volume was instilled using a pipette. MWCNTs (900–1201, lot-GS1802, SES Research, Houston, TX, United States) were freshly prepared and have been described previously (16). Sham controls received vehicle alone. Animals were sacrificed at 3, 10, 20, and 60 days post instillation and evaluated as previously described (4).

### Histological Analysis

Lungs were dissected and fixed in PBS-buffered 10% formalin. Paraffin embedded slides were sectioned at 7 μm, and stained with hematoxylin and eosin (H&E) or Gomori’s trichrome stain as previously described (4, 16). A previously described semiquantitative scoring system (7, 16, 17) was used to calculate a relative comparison of the numbers and quality of granulomas formed in MWCNT-instilled mice. Trichrome stain was evaluated using modified Ashcroft method (16, 18).

### Lymph Nodes

Tracheobronchial lymph node volume, identified based on Van den Broeck et al., was determined using the formula (Length × Width^2^)π/6, as previously described (16). Lymph nodes were fixed overnight in PBS-buffered 10% formalin and paraffin embedded. Representative Hemotoxylin/Eosin histological images were taken for each condition, time point and mouse strain using a Zeiss Axio Imager A1.

### Characterization of Bronchoalveolar Lavage Cells

BAL cells were obtained as previously described (15, 16). Total cell counts and differential counts were evaluated (Table 1). Cells were stored at −80°C for gene expression, and BAL fluid was aliquoted and frozen for protein analysis.

**TABLE 1:** BAL cell characteristics of C57Bl/6 and *Mmp12* KO mice 60 day.

	Treatment	*N*	Total cell count (×10^5^)	AM (X10^5^)	LYM (×10^5^)	PMN (×10^5^)
C57Bl/6	PBS/Surf	10	8.0 ± 2.9	7.7 ± 2.8 [97]	0.3 ± 0.3 [3]	0.06 ± 0.09 [1]
C57Bl/6	MWCNT	10	9.8 ± 2.5	8.9 ± 2.0 [92]	0.7 ± 0.5 [6]	0.2 ± 0.2 [2]
*Mmp12* KO	PBS/Surf	10	7.7 ± 2.5	7.5 ± 2.5 [97]	0.1 ± 0.1 [2]	0.05 ± 0.1 [1]
*Mmp12* KO	MWCNT	10	12.0 ± 4.1*	11.1 ± 4.0 [92]*	0.4 ± 0.2 [4]	0.5 ± 0.4 [4]*

AM, alveolar macrophages; LYM, lymphocytes; PMN, neutrophils. *Means ± SD, *p* ≤ 0.05 compared to PBS/Surf vs MWCNT in MMP12 KO. [ ] Percentage.

### RNA Purification and Gene Expression From BAL Cells

Total RNA was extracted from frozen BAL cells using miRNeasy Micro kit, (217084) (Qiagen, Germantown, MD, United States), according to manufacturer’s protocol. Mouse specific primers and probes were obtained from Qiagen, for *Mmp12* (PPM03619F), *Ccl2* (PPM03151G), *Ppar*γ (PPM05108B), and IFNγ (PPM03121A). GAPDH (PPM02946E) was used as a housekeeping gene. Quantitative-PCR was performed on complementary DNA synthesized with the RT2 First Strand Kit and evaluated on the StepOnePlus PCR system (Thermo Fisher Scientific, Waltham, MA, United States) in comparison to GAPDH using the 2^–Δ^ ^Δ^ ^CT^ method (19).

### Protein Analyses of BAL Fluid

CCL2 was assayed in BALF by ELISA ([MJE00B] R&D systems Minneapolis, MN, United States), as per manufacturer’s protocol.

### Immunostaining of BAL Cells and Frozen Tissue

Cytospin slides of BAL cells were fixed with 4% paraformaldehyde-PBS, permeabilized with Triton X-100, blocked and stained with anti-PPARγ at 1:250 dilution (Sc-7196) (Santa Cruz Biotechnology, Dallas, TX, United States), and Alexa 488 (1:1000) (Invitrogen, Carlsbad, CA, United States) or anti-IFNγ 1:200 (sc-57207) (Santa Cruz Biotechnology, Dallas, TX, United States) with secondary antibody Alexa Texas Red 569 (15).

Frozen lung tissue sections (7 μm) were fixed with 4% paraformaldehyde–PBS, permeabilized with Triton X-100, blocked with normal goat serum in PBS/Triton X-100 for nonspecific binding and stained with anti-MMP12 antibody (Sc-390863) (Santa Cruz Biotechnology, Dallas, TX, United States), 1:250 dilution, followed by Alexa conjugated goat anti-rabbit IgG 488 (Invitrogen, Carlsbad, CA, United States). Slides were counter-stained with DAPI (Vector Laboratories, Burlingame, CA, United States) to facilitate nuclear localization. Slides were imaged on Zeiss confocal LSM700.

### Statistical Analyses

Data were analyzed by Student’s *t*-test or one-way analysis of variance (ANOVA) using Prism 7 software (GraphPad, Inc., San Diego, CA, United States).

## Results

### MWCNT Instillation Increases MMP12 Expression in BAL Cells of Wild-Type Mice

**FIGURE 1:**
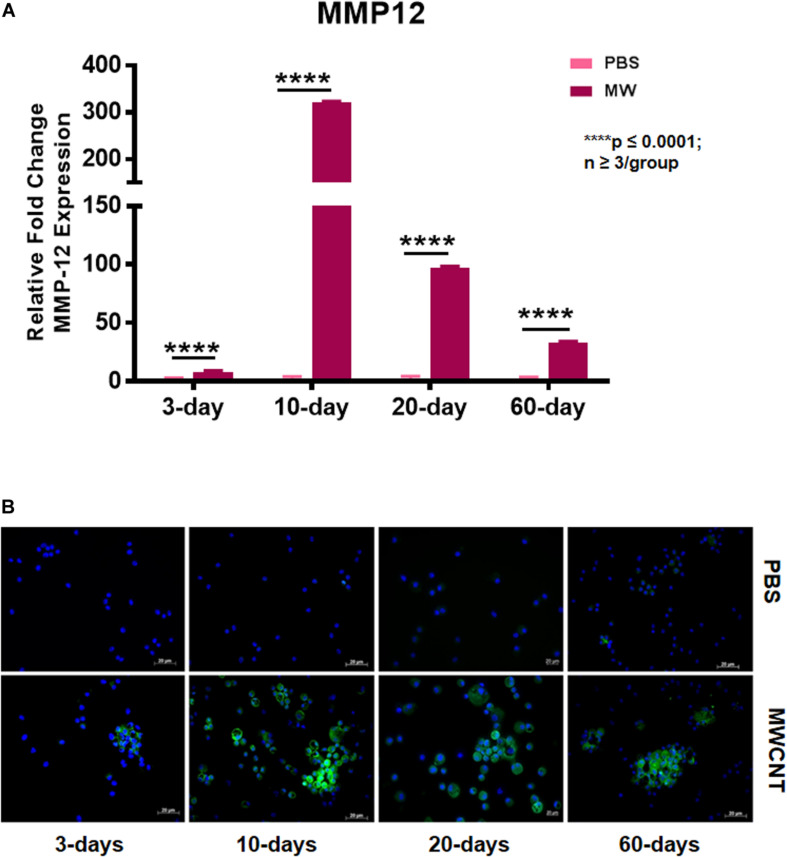
*Mmp12* gene expression and protein are upregulated in BAL cells of MWCNT-instilled C57BL/6. **(A)** *Mmp12* gene expression is increased at 3 days, with a peak at 10 days, and remains significantly upregulated at 60 days post MWCNT-instillation (*p* ≤ 0.05; *n* ≥ 3 per group). **(B)** Immunofluorescent anti-MMP12 staining was minimal at 3 days, with a peak at 10 days and persistence through 60 days (representative of *n* = 3).

**FIGURE 2:**
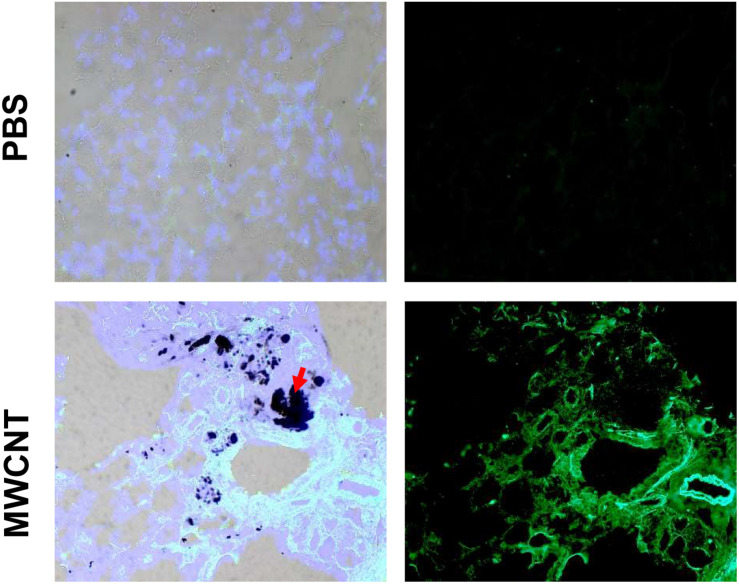
MMP12 protein is upregulated in pulmonary granulomas of 60-day MWCNT-instilled C57BL/6 mice. Representative bright-field and immunofluorescence images for MMP12 protein indicate very minimal to no staining in PBS instilled mice, but increased protein around granulomas in MWCNT-instilled C57BL/6 at 60-days. Black aggregates in (lower left, red arrows) bright-field images are MWCNT accumulations in the tissue.

To explore MMP12 involvement in granuloma development, we examined the time course of *Mmp12* expression. C57BL/6J wildtype mice were instilled with MWCNT and BAL cells were collected after 3, 10, 20, and 60 days. Quantitative RT-PCR (qRT-PCR) of BAL cells revealed significant increases in *Mmp12* mRNA expression at 3 days (7.6-fold) with a peak at 10 days (322-fold) and sustained elevation at 60 days (33-fold) when compared to PBS-instilled mice (sham controls) (Figure 1A). Similarly, immunofluorescence showed MMP12 protein expression to be upregulated in wildtype BAL cells (Figure 1B). MMP12 appeared most prominent at 10 days post instillation and persisted to 60 days. In lung tissues from wildtype animals, expression of MMP12 protein was also upregulated around granulomas at 60-days after MWCNT instillation (Figure 2).

### Progression of Granuloma Formation Is Attenuated in MWCNT-Instilled *Mmp12* KO Mice

**FIGURE 3:**
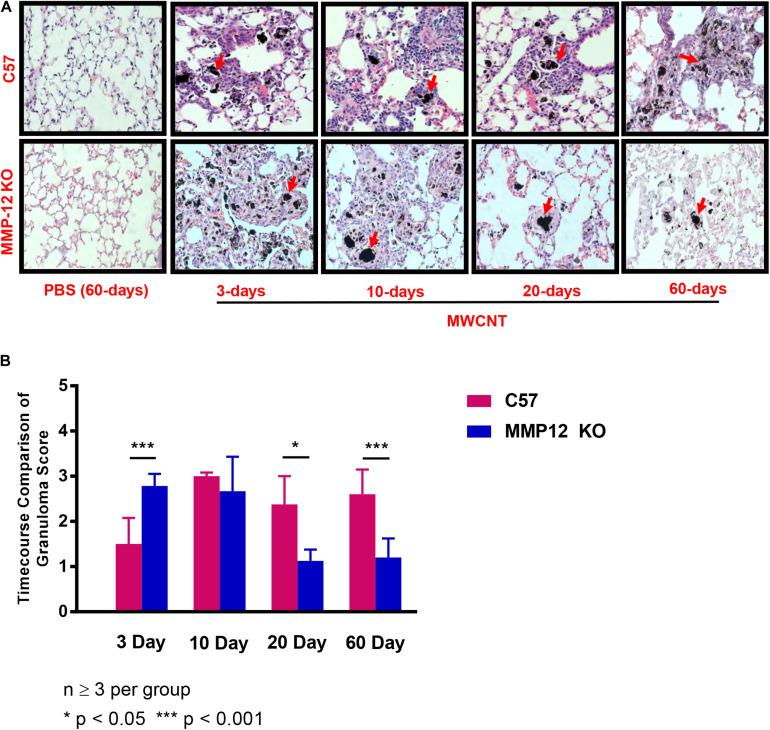
Granulomas from *Mmp12* KO mice are resolving at 60 days. **(A)** Histological examination of H&E-stained lung tissue sections from MMCNT-instilled C57BL/6 and *Mmp12* KO mice indicate loosely formed granulomas as early as 3 days in *Mmp12* KO mice with resolution beginning at 20 days in contrast to C57BL/6. Representative bright field images illustrating granuloma formation in MWCNT-instilled mice at 60 days show decreased size and number of granulomas in *Mmp12* KO compared to C57BL/6 mice. Red arrows indicate deposition of MWCNT in the lung. **(B)** Granuloma scoring (mean ± SEM) of *Mmp12* KO as compared to C57BL/6. *Mmp12* KO lung scores were significantly less than those of C57BL/6 at 60 days (***p < 0.001). Sections were evaluated by two independent observers from *n* = 6 lungs for each group.

The effects of MMP12 on granuloma formation were examined in *Mmp12* KO and wildtype mice instilled with MWCNT. Pulmonary histological changes were observed in both *Mmp12* KO and wildtype mice at 3, 10, 20, and 60 days post MWCNT instillation compared to PBS controls (Figure 3A). Granulomas formed in wildtype mice were detected as early as 10 days post instillation. These early granulomas were poorly formed, but by 60 days post instillation, granulomas appeared to be well defined. Surprisingly, no histological differences in granuloma formation were noted acutely in *Mmp12* KO mice compared to wildtype at days 3 and 10. In contrast, by 20 days after MWCNT instillation, granulomas in *Mmp12* KO mice appeared to be resolving and by 60 days were smaller and less well-formed. None of the time points showed evidence of necrosis or caseation. Histological analyses at 60-days post instillation (Figure 3B) were scored based upon size and frequency of granulomas. Scores were significantly (*p* = 0.01) less in *Mmp12* KO mice. Trichrome staining revealed no fibrosis in MWCNT-instilled *Mmp12* KO mice (data not shown).

### Mediastinal Lymphadenopathy Is Attenuated in *Mmp12* KO Mice at 60 Days

**FIGURE 4:**
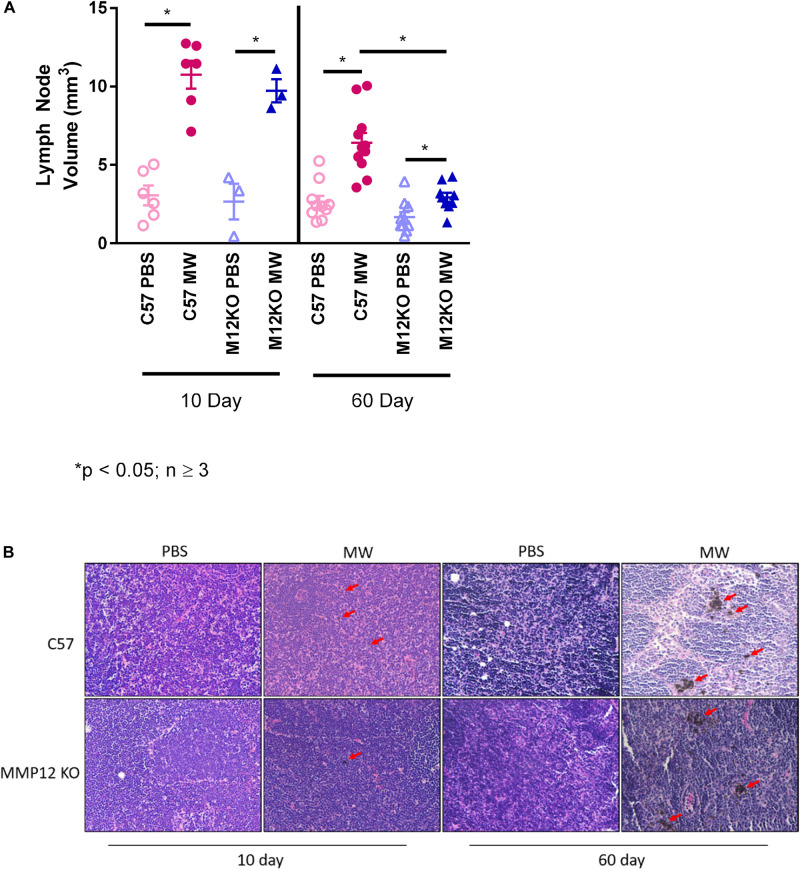
Mediastinal Lymph node volume is decreased at 60-days in MWCNT-instilled *Mmp12* KO mice. **(A)** At 10 days lymph node volumes were not significantly different between MWCNT-instilled C57BL/6 and *Mmp12* KO mice. However, at 60 days, lymph node volumes from MWCNT-instilled *Mmp12* KO mice were significantly decreased compared to C57BL/6 (*p ≤ 0.05, *n* ≥ 3/group). **(B)** Carbon particle deposition can be seen in both C57BL/6 and *Mmp12* KO mice at 10 days (red arrows) and is more prominent at 60-day (red arrows) as compared to respective lymph nodes from PBS-instilled mice.

MWCNT promoted an exacerbated lymphadenopathy in wildtype mice (Figure 4A). We hypothesized that as granulomas in *Mmp12* KO mice were diminished at 60 days, mediastinal lymphadenopathy would also be attenuated in these mice. As predicted, MWCNT-instilled *Mmp12* KO mice exhibited reduced mediastinal lymph node sizes compared to wildtype at 60 days after instillation (Figure 4). It should be noted that at 10 days, there were no differences in mediastinal lymph node volume between wildtype and *Mmp12* KO mice. Granulomas were not present in the lymph nodes of either wildtype or *Mmp12* KO mice (Figure 4B). However, MWCNT are present in wildtype and *Mmp12* KO mice at 10 days and MWCNT deposition is increased in both at 60 days.

### MWCNT-Instilled *Mmp12* KO Mice at 60 Days Have Increased Macrophage Influx in BAL

Surprisingly, despite the resolution of granulomatous inflammation at 60 days, MWCNT-instilled *Mmp12* KO mice had significantly (*p* ≤ 0.05) increased numbers of macrophages in BAL fluid compared to *Mmp12* PBS controls or MWCNT-instilled wildtypes (Table 1). These results suggest that macrophage influx may be involved in the resolution of granulomatous inflammation.

### MMP12 Deficiency Does Not Affect CCL2 Expression in BAL Cells and Fluids

**FIGURE 5:**
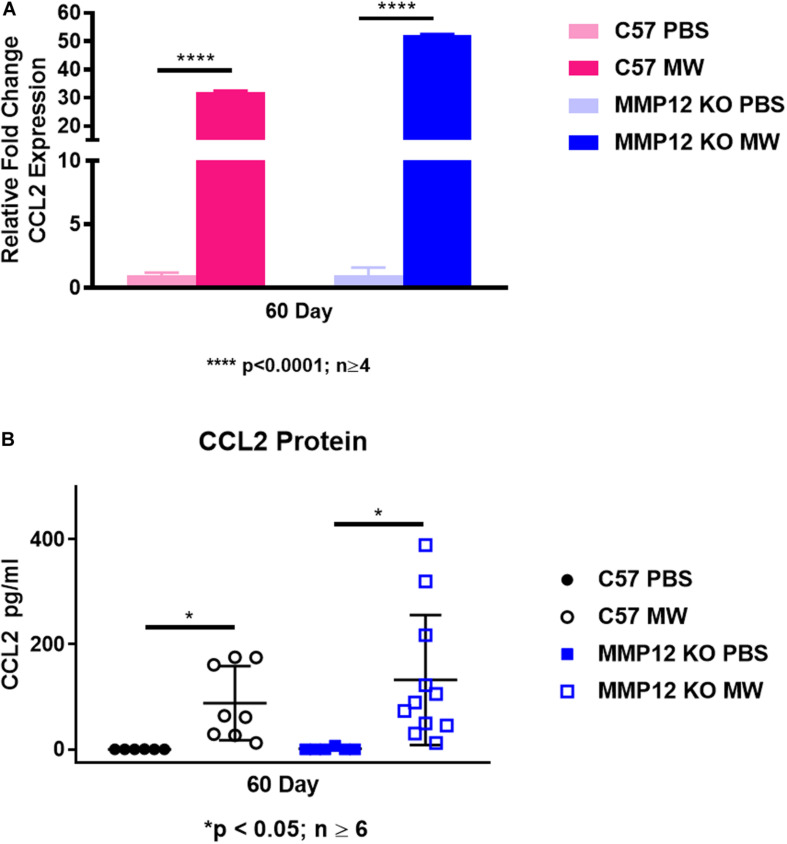
*Ccl2* gene expression and protein are elevated in both C57BL/6 and *Mmp12* KO mice at 60 days after MWCNT instillation. **(A)** *Ccl2* gene expression is increased in BAL cells of both C57BL/6 and *Mmp12* KO MWCNT-instilled mice compared to PBS controls (****p < 0.0001; *n* ≥ 4). **(B)** CCL2 protein from the BAL fluid is increased in both C57BL/6 and *Mmp12* KO MWCNT-instilled mice compared to PBS (*p ≤ 0.05; *n* ≥ 6).

Because of the increased number of macrophages in BAL fluid of MWCNT-instilled *Mmp12* KO mice at 60 days despite the granuloma resolution, we investigated the monocyte/macrophage chemokine CCL2. Unexpectedly, *Ccl2* gene expression in BAL cells was not different in MWCNT-instilled *Mmp12* KO mice compared to wildtype despite the histological resolution (Figure 5A). In order to determine whether CCL2 was elevated in alveolar spaces, BAL fluid was analyzed. CCL2 protein was elevated in BAL fluids from MWCNT-instilled *Mmp12* KO and wildtype mice compared to PBS-instilled controls and increased levels did not differ between the two mouse strains (Figure 5B).

### PPARγ Expression Is Increased in *Mmp12* KO Mice Compared to Wild Type

**FIGURE 6:**
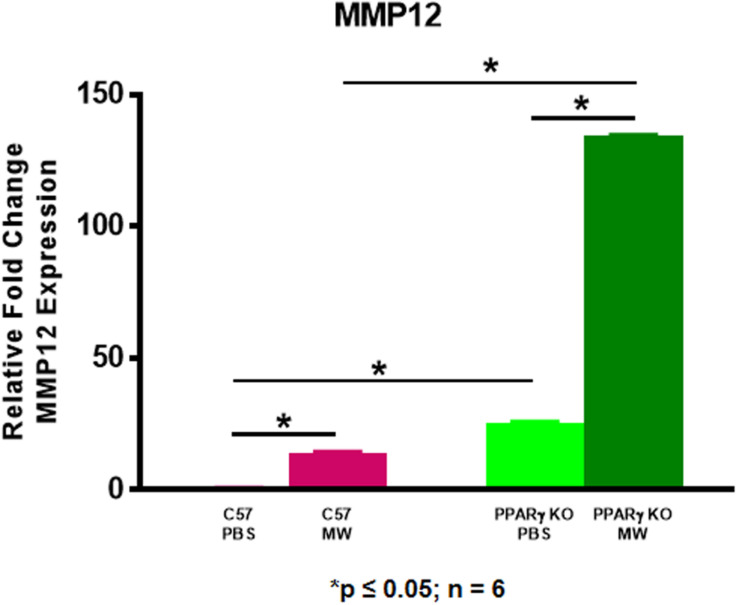
MMP12 is elevated in MWCNT-instilled *Ppar*γ KO BAL cells. *Mmp12* gene expression in *Ppar*γ KO mice is significantly increased intrinsically, and further increased after MWCNT instillation compared to C57BL/6 (*p ≤ 0.05; *n* = 6/group).

**FIGURE 7:**
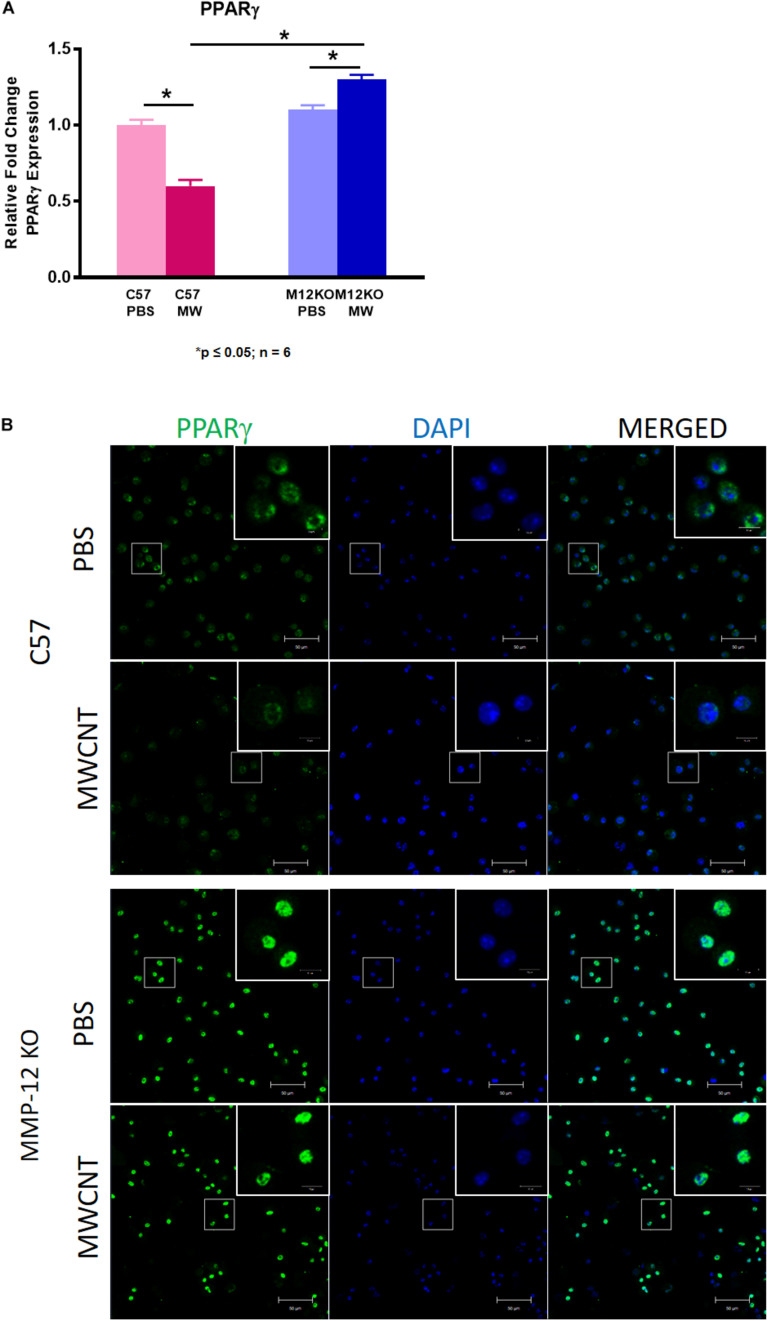
*Ppar*γ gene expression and protein are increased in 60-day MWCNT-instilled *Mmp12* KO mice compared to C57BL/6. **(A)** *Ppar*γ gene expression is decreased in MWCNT-instilled C57BL/6 mice compared to PBS controls. In contrast, *Ppar*γ is significantly increased in MWCNT-instilled *Mmp12* KO mice compared to PBS controls (*p ≤ 0.05; *n* ≥ 6 /group). **(B)** Immunofluorescent anti-PPARγ staining of BAL cells from PBS-instilled mice show constitutive PPARγ protein as indicated by green fluorescence, while MWCNT-instilled C57BL/6 show decreased expression. PBS instilled *Mmp12* KO mice show intrinsically high levels of PPARγ protein, and with MWCNT, PPARγ protein remains highly expressed (representative figure of *n* = 3).

We postulated that MMP12 levels might be higher in PPARγ KO mice. As shown in Figure 6, MMP12 expression was significantly elevated in BAL cells of PBS-instilled *Ppar*γ KO mice and further increased after MWCNT instillation compared to wildtype. These findings suggested a PPARγ regulatory role in MMP12 expression. Based on these data, we hypothesized that PPARγ might be elevated in BAL cells from *Mmp12* KO mice since granuloma formation was decreased. At 60 days, *Mmp12* KO BAL cells from MWCNT-instilled mice exhibited elevated PPARγ expression in contrast to wild-type where PPARγ was decreased (Figure 7A). In order to confirm whether PPARγ was active in *Mmp12* KO mice, BAL cells were stained with anti-PPARγ antibody. Wildtype mice instilled with MWCNT exhibited decreased PPARγ protein. PBS-instilled *Mmp12* KO mice showed increased PPARγ protein, which further increased after MWCNT instillation (Figure 7B).

### IFN-γ Gene and Protein Expression Are Not Increased in MWCNT-Instilled *Mmp12* KO Compared to Wild Type

**FIGURE 8:**
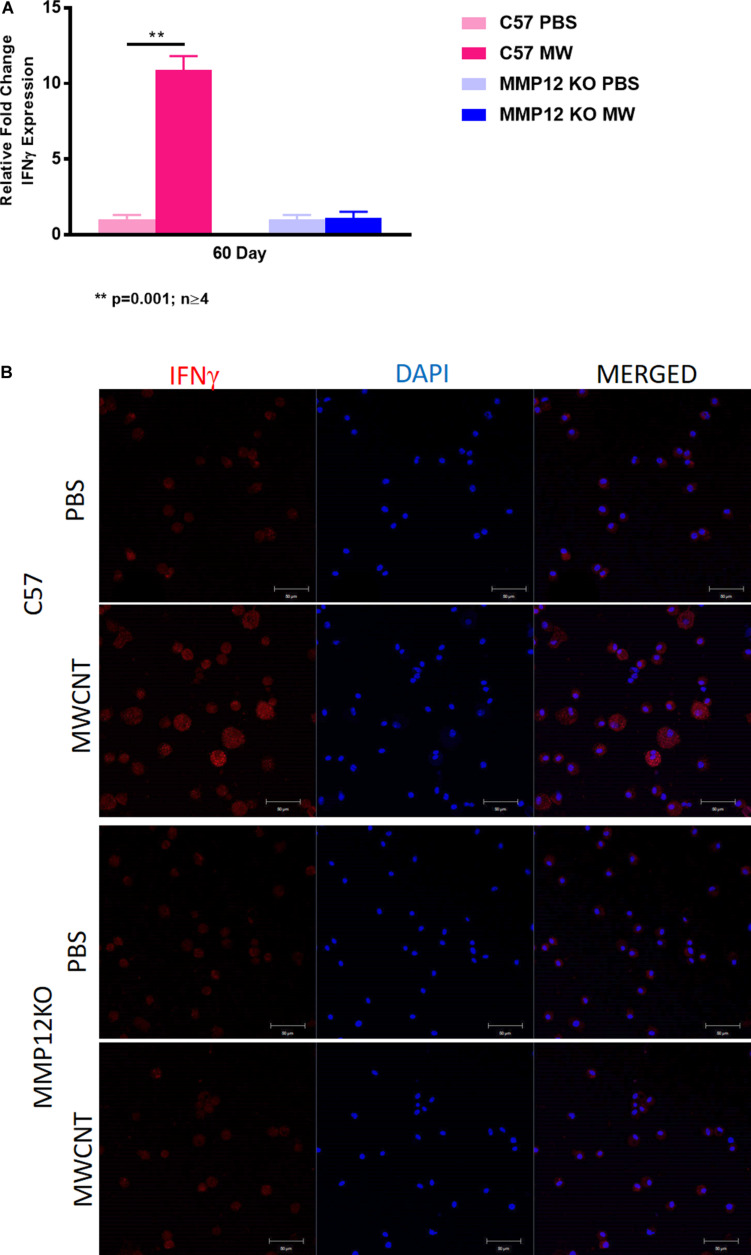
IFNγ gene expression and protein are not increased in MWCNT-instilled *Mmp12* KO compared to C57BL/6. **(A)** *Ifn*γ gene expression is significantly (**p = 0.001; *n* ≥ 4) increased in C57BL/6 mice instilled with MWCNT compared to PBS controls. In *Mmp12* KO mice, *Ifn*γ gene expression is not increased by MWCNT instillation compared to PBS controls. **(B)** Immunofluorescent anti-IFNγ staining of BAL cells from PBS-instilled mice show minimal IFNγ protein, while MWCNT-instilled C57BL/6 show marked IFNγ protein as indicated by red fluorescence. Both PBS and MWCNT-instilled *Mmp12* KO mice showed minimal staining for IFNγ protein (representative figure of *n* = 3).

We investigated IFN-γ expression in the MWCNT murine model and found that IFN-γ gene expression was decreased in MWCNT-instilled *Mmp12* KO mice compared to wild type (Figure 8A). IFN-γ protein was evaluated by immunostaining of BAL cytospins to confirm the differences. MWCNT-instilled wildtype mice demonstrated prominent IFN-γ protein expression. In contrast MWCNT-instilled *Mmp12* KO mice exhibited almost no detectable IFN-γ (Figure 8B).

## Discussion

The current findings in the murine MWCNT granuloma model highlight the importance of MMP12 in granuloma pathophysiology and complement findings in sarcoidosis. Our previous and current reports demonstrate that inflammation and granuloma formation in the MWCNT model are associated with a marked increase in MMP12 expression as early as 10 days post instillation with a significant increase persisting to 60 days (4). These data suggest that MMP12 is required for the chronic stages of inflammation associated with granuloma formation. In sarcoidosis, MMP12 constitutes one of the most highly expressed genes in granulomatous lung tissues (12). Our previous comparative transcriptional survey of alveolar macrophages from sarcoidosis patients and MWCNT-instilled mice also revealed marked MMP12 elevation in both species (13). Numerous previous studies have also shown that MMP12, an elastase enzyme predominantly produced by macrophages, is an important mediator of both acute and chronic lung injury and directly involved in development of inflammatory responses (9, 10). MMP12 mechanisms involve degradation of the extracellular matrix protein, elastin, into fragments which can act as a chemoattractant for macrophage recruitment (20).

The monocyte/macrophage chemokine CCL2 has been linked to granuloma formation in other animal model systems (21) and transcriptional studies also indicated elevated expression in both MWCNT instilled mice and sarcoidosis patients (13, 14). Elevation of CCL2 in BAL cells was unexpected in *Mmp12* KO mice at 60 days after MWCNT instillation when granulomas had resolved. In our previous studies, CCL2, which is produced by macrophages, was consistently elevated in parallel with MWCNT-induced granuloma formation (4, 7). Upregulation of CCL2 in BAL fluid is also characteristic of patients with pulmonary sarcoidosis (22, 23). We noted that an influx of BAL macrophages accompanied the granuloma resolution in *Mmp12* KO mice at 60 days. Whether this is driven by CCL2 is unclear. Recent studies have suggested that CCL2 functions may extend beyond its original characterization as a chemoattractant [reviewed in Gschwandtner et al. (24)]. Additional functions attributed to CCL2 have included adhesion, polarization, and effector molecule secretion, and many are context-dependent and may be synergistic with other inflammatory stimuli (24). Gene expression analyses in both MWCNT-instilled mice and sarcoidosis indicated a multitude of elevated inflammatory mediators that may modify CCL2 effects (12, 13). Additional studies will be required to define the complex role of CCL2 in MMP12 regulation.

The current findings also noted a lack of IFN-γ upregulation in 60-day MWCNT-instilled *Mmp12* KO mice in contrast with wildtypes in which IFN-γ was significantly increased as noted in previous MWCNT studies (7, 25). Elevated IFN-γ in sarcoidosis patients with pulmonary disease has been well-reported in the literature (26, 27, 28). Upregulated expression of IFN-γ signaling pathways was also found in both MWCNT-instilled wildtype mice and sarcoidosis patients in our recent transcriptional survey of alveolar macrophages from both groups (13, 14). Thus, the current studies emphasize an association between IFN-γ and granulomatous changes in the lung. In contrast to absence of IFN-γ, expression of PPARγ was elevated in *Mmp12* KO mice at 60 days post MWCNT instillation. PPARγ is a nuclear receptor that regulates expression of genes involved in lipid homeostasis and inflammation in immune cells especially macrophages (29, 30). Interestingly, PPARγ and IFN-γ exhibit mutually antagonistic properties (8, 31) which may explain, in part, our observations. However, further studies are needed to better elucidate the interconnected role of IFN-γ – PPARγ pathways in *Mmp12* KO mice.

Our previous studies demonstrated that PPARγ deficiency exacerbates granuloma formation in the MWCNT murine granuloma model (7). The relationship between PPARγ and MMP12 in granuloma formation has not been previously described, and the current data show for the first time an inverse relationship between PPARγ and MMP12 in mediating pulmonary granulomatous inflammation. MMP12 expression is increased by PPARγ deficiency as shown by the MWCNT experiments in PPARγ KO mice. In *Mmp12* KO mice, both PPARγ expression and activity increased with MWCNT instillation, suggesting that MMP12 deficiency enhances PPARγ. Overall, our data suggest that PPARγ pathways may contribute to the reduction of granuloma formation in *Mmp12* KO mice.

**FIGURE 9:**
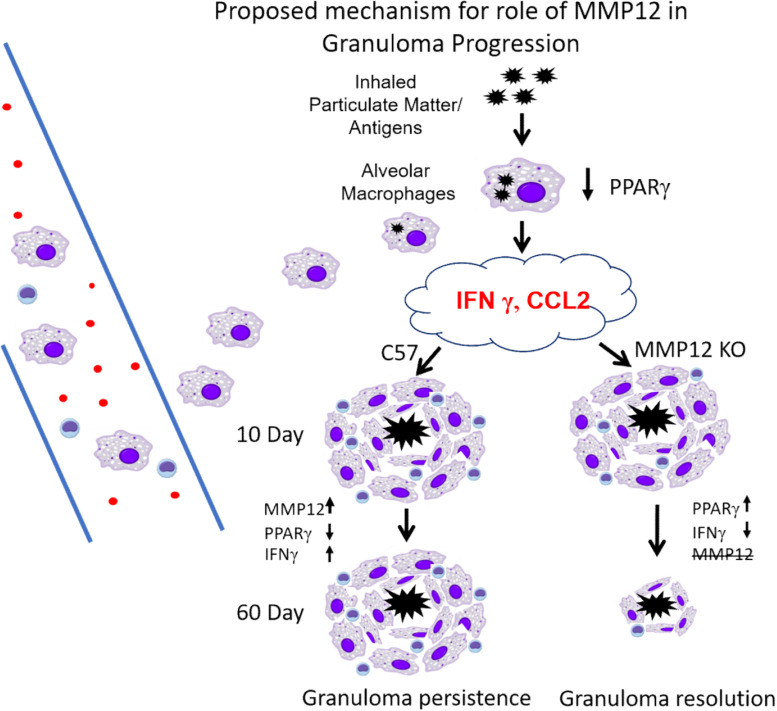
Proposed mechanism for role of MMP12 in granuloma progression. Initial exposure to inhaled particulate matter/and or antigens triggers secretion of several cytokines (CCL2, IFNγ), with further recruitment of alveolar macrophages/monocytes and T cells. Once on site, macrophages produce additional cytokines which promote the formation of multinucleated giant cells and retention of T cells. With MMP12 upregulation (C57/Bl6) persistent PPARγ decrease and IFNγ increase results in granuloma persistence. Whereas as in the absence of MMP12 (*Mmp12 KO*) PPARγ increases and IFNγ decreases resulting in granulomas resolution.

Evidence from the present study and previous studies (4, 7, 16, 25) is summarized in Figure 9. We propose that an initial exposure to inhaled particulate matter and/or various antigens (which may include bacterial components such as mycobacterial peptides) triggers the alveolar macrophage secretion of several cytokines (CCL2, IFNγ) with further recruitment of alveolar macrophages/monocytes and T cells. Once on site, macrophages produce additional cytokines which promote the formation of multinucleated giant cells and retention of T cells. With MMP12 upregulation (C57/Bl6), persistent PPARγ decrease and IFNγ increase results in granuloma persistence. Whereas in the absence of MMP12 (*Mmp12 KO*), PPARγ increases and IFNγ decreases, resulting in granuloma resolution.

Decades of research suggest that the etiology of sarcoidosis may be multifactorial and complex, as illustrated by the multiple environmental factors which have been associated with sarcoidosis disease development [reviewed by Judson (32)]. Studies of lung tissues from sarcoid patients have found silica, aluminum, and titanium as well as carbon nanotubes (33). Fibrotic granulomatous lung disease together with elevated CCL2 and MMP12 gene expression as we have described in the wildtype murine MWCNT model have been reported in a rat model of chronic silicosis (34). This model, however, lacks some key sarcoidosis features such as elevated IFN-γ and also does not exhibit any MMP12 protein, unlike the MWCNT model in which high levels of MMP12 protein were detected in both alveolar macrophages and lung tissues from MWCNT-instilled mice. A recent review of available animal models of sarcoidosis concluded that no single model faithfully reproduces all aspects of sarcoidosis pathology (35), but the models may help in evaluating selective sarcoidosis pathways that can be reproduced in the models.

## Conclusion

This study demonstrates that MMP12 deficiency reduces pulmonary granuloma progression and highlights a critical role for MMP12 in the chronicity of granulomatous inflammation. Potential mechanisms involved in granuloma resolution require further exploration, including identifying how the down-regulation of IFN-γ results in elevated PPARγ, and a better understanding of how CCL2 promotes macrophage recruitment. Deciphering MMP12–orchestrated mechanisms in granuloma formation can lead to novel approaches for treating sarcoidosis.

## Data Availability Statement

The raw data supporting the conclusions of this article will be made available by the authors, without undue reservation, to any qualified researcher.

## Ethics Statement

The animal study was reviewed and approved by IACUC of East Carolina University.

## Author Contributions

NN, ES, MM, NL, DO, and DT: acquisition of data. AMo, AMa, WK, BB, SG, LS, and MJT: concept and design. BB, AMa, and MJT: analysis, interpretation, and drafting of manuscript for important intellectual content. All authors contributed to the article and approved the submitted version.

## Conflict of Interest

The authors declare that the research was conducted in the absence of any commercial or financial relationships that could be construed as a potential conflict of interest.

## Footnotes

1 https://www.ncbi.nlm.nih.gov/geo
